# Role of the sigma-1 receptor chaperone in rod and cone photoreceptor degenerations in a mouse model of retinitis pigmentosa

**DOI:** 10.1186/s13024-017-0202-z

**Published:** 2017-09-19

**Authors:** Huan Yang, Yingmei Fu, Xinying Liu, Pawan K. Shahi, Timur A. Mavlyutov, Jun Li, Annie Yao, Steven Z.-W. Guo, Bikash R. Pattnaik, Lian-Wang Guo

**Affiliations:** 1 Department of Surgery, Wisconsin Institute for Medical Research, University of Wisconsin School of Medicine and Public Health, Madison, WI 53705 USA; 2 Shanghai Key Laboratory of Psychotic Disorders, Shanghai Mental Health Center, Shanghai Jiao Tong University School of Medicine, 600 Wanping Nan Road, Shanghai, 200030 People’s Republic of China; 3 Department of Pediatrics, Department of Ophthalmology and Visual Sciences, University of Wisconsin School of Medicine and Public Health, 1300 University Avenue, SMI 112, Madison, WI 53706 USA; 4 Department of Anesthesiology, Wisconsin Institute for Medical Research, University of Wisconsin School of Medicine and Public Health, Madison, WI 53705 USA; 5 Department of Ophthalmology, the First Hospital of China Medical University, Shenyang, 110001 People’s Republic of China; 6 Department of Ophthalmology, the 3rd People’s Hospital of Dalian, Dalian, 116033 People’s Republic of China; 7 McPherson Eye Research Institute, University of Wisconsin, Madison, WI 53705 USA; 8 Department of Surgery and Department of Physiology &Cell Biology, the Ohio State University, Columbus, OH 43210 USA

**Keywords:** The sigma-1 receptor, rd10/S1R^−/−^ and rd10/S1R^+/+^ mice, necroptosis, autophagy, electroretinography (ERG)

## Abstract

**Background:**

Retinitis pigmentosa (RP) is the most common inherited retinal degenerative disease yet with no effective treatment available. The sigma-1 receptor (S1R), a ligand-regulated chaperone, emerges as a potential retina-protective therapeutic target. In particular, pharmacological activation of S1R was recently shown to rescue cones in the rd10 mouse, a rod *Pde6b* mutant that recapitulates the RP pathology of autonomous rod degeneration followed by secondary death of cones. The mechanisms underlying the S1R protection for cones are not understood in detail.

**Methods:**

By rearing rd10/S1R^−/−^ and rd10/S1R^+/+^ mice in dim light to decelerate rapid rod/cone degeneration, we were able to compare their retinal biochemistry, histology and functions throughout postnatal 3–6 weeks (3 W–6 W).

**Results:**

The receptor-interacting protein kinases (RIP1/RIP3) and their interaction (proximity ligation) dramatically up-regulated after 5 W in rd10/S1R^−/−^ (versus rd10/S1R^+/+^) retinas, indicative of intensified necroptosis activation, which was accompanied by exacerbated loss of cones. Greater rod loss in rd10/S1R^−/−^ versus rd10/S1R^+/+^ retinas was evidenced by more cleaved Caspase3 (4 W) and lower rod electro-retinographic a-waves (4 W–6 W), concomitant with reduced LC3-II and CHOP (4 W–6 W), markers of autophagy and endoplasmic reticulum stress response, respectively. However, the opposite occurred at 3 W.

**Conclusion:**

This study reveals previously uncharacterized S1R-associated mechanisms during rd10 photoreceptor degeneration, including S1R’s influences on necroptosis and autophagy as well as its biphasic role in rod degeneration upstream of cone death.

**Electronic supplementary material:**

The online version of this article (doi:10.1186/s13024-017-0202-z) contains supplementary material, which is available to authorized users.

## Background

The sigma-1 receptor (S1R) is a molecular chaperone that modulates a variety of cellular activities. While ubiquitously distributed, it is abundant in the central and peripheral nervous systems [1]. The S1R binding site is the target of numerous pharmacological studies on psychotic disorders [2], locomotor activity [3], threat response [4], and pain [5] etc. Many S1R-binding compounds have been identified [6]; some are in clinical use (e.g., as antidepressants) [7] or trials [8]. A protective role of S1R has been increasingly reported in neurodegenerative disease models [7], including Parkinson’s [9], Alzheimer’s [10], Huntington’s [11] and ALS [12]. Furthermore, human S1R mutations were linked to ALS [13] and frontotemporal lobar degeneration [14]. Abnormal S1R subcellular localization was found in postmortem brain samples of neurodegenerative diseases [15]. Thus, S1R appears to be a potential anti-neurodegenerative therapeutic target. The recently solved S1R crystal structure [16] is expected to accelerate therapeutic development of S1R drugs.

Degeneration of retinal neurons, mainly photoreceptors (PRs, i.e., rods and cones) and retinal ganglion cells (RGCs), shares considerable patho-mechanisms with neurodegenerative brain diseases. An important recent development is that S1R was found to protect RGCs (for review, see [17, 18]), in RGC-damage mouse models either treated with S1R agonists [19, 20] or compared between wild type and S1R knockout (S1R^−/−^) [21, 22]. Wang et al. [23] just reported that intraperitoneal repeat injections of S1R agonist (+)-pentazocine substantially rescued cones in the rd10 mouse model (rod *Pde6b* mutation) of retinitis pigmentosa (RP). RP is a heterogeneous group of inherited retinal degenerations linked to thousands of mutations in over 70 human genes [24]. The rd10 model exemplifies RP with autonomous rod degeneration and secondary cone death [25]. As it is not yet practical to correct RP mutations individually, investigation of a common pathway, e.g., S1R, a target suitable for pharmacological interventions [6], is highly significant. It is therefore imperative to understand how S1R influences PR death in rd10 mice.

A recent report distinguishes that apoptosis and necroptosis (programed necrosis) are chief mechanisms responsible for the sequential death of rods and cones, respectively [25]. While apoptosis has been well-documented, necroptosis of cones is a relatively new mechanism not well understood in rd10 retinas. In particular, whether S1R influences this process is not known. Moreover, since cone death results from dying rods in rd10 retinas [25], it is important to understand whether the S1R protection against cone death at later stages involves its influence on rod degeneration at earlier stages. However, the specific role(s) of S1R in rod degeneration remains unclear, presumably because the previous study primarily focused on cone death [23], a stage when rod function was barely detectable.

To investigate the specific role(s) of S1R at different stages of rd10 PR degeneration, we designed a unique experimental setting with the following considerations. First, we crossed rd10 to S1R knockout mice [3] and used this S1R-null strain (rd10/S1R^−/−^) throughout to compare with rd10 mice (rd10/S1R^+/+^). Second, we reared these mice in dim red light (< 5 lx) instead of regular housing light to retard aggressive rd10 PR degeneration, so that we were able to improve the “temporal resolution” of our data. Third, we studied the impact of S1R knockout on the activation of necroptosis, the mode of cone death [25]. Fourth, we determined the time course including early time points to define the role of S1R in rod degeneration that precedes cone death. We observed a dramatic increase of necroptosis activation in rd10/S1R^−/−^ retinas compared to rd10/S1R^+/+^ retinas, and also made a surprising finding that in early stage rods were protected without S1R. The underlying mechanisms are discussed.

## Methods

### Animal ethics statement

All animal procedures conformed to the NIH guide for the ethical care and use of laboratory animals and were in compliance with the ARVO Statement for the Use of Animals in Ophthalmic and Vision Research. Animal protocols were approved by the Institutional Animal Care and Use Committee of University of Wisconsin-Madison and Ohio State University. All surgeries were performed under isoflurane anesthesia. Animals were maintained on a 4% fat diet (8604 M/R, Harkland Teklad, Madison, WI), and euthanized in a chamber gradually filled with CO_2_.

### Mouse strains, breeding, and rearing

A homozygous rd10 mouse breeding pair (B6.CXB1-*Pde6b* ^*rd10*^/J, stock number 004297) were purchased from the Jackson Laboratory (Bar Harbor, ME). This model of retinitis pigmentosa (RP) is a spontaneous missense point mutation in *Pde6b* (rod-specific cGMP phosphodiesterase 6 beta subunit) [26]. Progressive rod (and then cone) photoreceptor degeneration in homozygous rd10 mice begins at postnatal day 16 and completes at day 35.

A breeding pair of heterozygous S1R knockout mice, which are Oprs1 mutant (+/−) Oprs^Gt(IRESBetageo)33Lex^ on a C57BL/6 J × 129 s/SvEv mixed background, were purchased from the Mutant Mouse Regional Resource Center (UC Davis, CA). The colony of homozygous S1R knockout mice (S1R^−/−^) was established after back-crossing to C57BL/6 J mice for nine times to reach a relatively uniform background. The S1R^−/−^ mice were then crossed with rd10 mice to generate an rd10/S1R^−/−^ strain. Litter mates of rd10/S1R^−/−^ and rd10/S1R^+/+^ mice were confirmed by genotyping (see below) and then used for experiments. In order to slow down photoreceptor degeneration thus enlarging the window of experimental time course, these mice were reared in a dedicated room equipped with dim (<5 lx) red light (12 h/12 h light/dark). The rearing conditions were otherwise standard.

Homozygous Nrl-GFP mice, or B6.Cg-Tg(Nrl-EGFP)1Asw/J (stock number 021232), were purchased from the Jackson Laboratory (Bar Harbor, ME). In this transgenic strain, the *Nrl* (neural retina leucine zipper gene) promoter drives expression of EGFP specifically in rod photoreceptors and the pineal gland. In the adult retina, GFP is detected only in the outer nuclear layer, which contains rod and cone photoreceptor nuclei, and in the corresponding rod inner and outer segments.

### Genotyping of rd10/S1R^−/−^ and rd10/S1R^+/+^ mice

For S1R genotyping, primers 5′-tctgagtacgtgctgctcttcg (985–5′), 5′-cagaaatctcagcccagtatcg (985–3′) and 5′-ataaaccctcttgcagttgcatc (LTR-rev) were synthesized. The pair of 985–5′ and 985–3′ were used to amplify the *Sigmar1* wildtype allele (~200 bp PCR products), and the pair of 985–5′ and LTR-rev were used to identify the *Sigmar1* knockout allele (~400 bp PCR products) [3]. To genotype rd10, primers 5′-ctttctattcctctgtcagcaagc (Pde6b13F) and 5′-catgagtagggtaaacatggtctg (Pde6b13R) were used [26]. After PCR amplification, products (97 bp) were subjected to 2 ~ 5 units of *Cfo*I (Promega) digestion for 2 h at 37 °C. While the non-cleavable *Pde6b* wildtype products showed a size of 97 bp, the *Pde6b* ^*rd10*^ mutant products were processed into fragments of 54 bp and 43 bp.

### Preparation of retinal sections and homogenates

In this study, the majority of experiments were performed for a time course spanning from postnatal 3 weeks (3 W) to 6 W. Mice were euthanized by CO_2_ asphyxiation followed by cervical dislocation at 3 W, 4 W, 5 W, and 6 W. Eyeballs were enucleated immediately and dissected. For morphometric and immunohistochemistry analyses, retinal cryosections were prepared according to our published methods [21, 27]. Briefly, eyeballs were fixed in 4% paraformaldehyde for 7 h at 4 °C and then soaked in 30% sucrose in PBS for 14 h at 4 °C, and 10 μm sections were cut from the eyeballs frozen in optimum cutting temperature (OCT) embedding medium (Sakura Finetek USA, Inc., Torrance, CA). For Western blotting, unfixed eyeballs were used. The cornea was removed, and the retina was carefully dissected out of the eyecup. Caution was taken to avoid contamination of the retinal pigment epithelium (RPE). Retinas were homogenized and immediately used for protein separation on a polyacrylamide gel followed by immunoblotting [27].

### Immunohistochemistry and fluorescence microscopy

Immunostaining was performed on retinal cryosections following our previously described method [28] with minor modifications. Briefly, retinal sections were permeabilized with 0.1% Triton X-100 in PBS for 20 min, blocked with 5% normal donkey serum (017–000-121; Jackson Immunoresearch Lab, MS) for 1 h at room temperature, and then incubated with a primary antibody overnight at 4 °C. Sources and dilutions of primary antibodies are the following: Anti-ACTIVE® Caspase-3 pAb (Promega, G7481), 1:250, Rabbit anti-IBA-1 (Wako, 019–19,741), 1:100; Rabbit anti-GFAP (Cell Signaling, 12,389), 1:100; rabbit anti-S1R (in-house produced), 1:50; Mouse anti-BRN3A (Millipore, AB5945), 1:100. After rinsing the section 3×, a secondary antibody (Alexa-488 conjugated donkey-anti-rabbit or Alexa-555-conjugated donkey-anti-mouse) at 1 μg/ml was applied at room temperature for 1 h. Sections stained with a secondary antibody, but not a primary antibody, were used for negative control. Sections were then rinsed 3×, counterstained with Hoechst 33,342 (Life Technologies, H3570) at 5 μg/ml for 1 min, and then mounted in Prolong Gold mounting medium (Invitrogen, Carlsbad, CA) and cover-slipped. The slides were left in the dark overnight and then sealed using clear nail polish (Electron Microscopy Sciences, Hatfield, PA). To visualize cones, retinal sections were incubated with Fluorescein-labeled Peanut Agglutinin (PNA, Vector Laboratories, FL-1071, 1:500 dilution) for 1 h at room temperature and then washed. Images were acquired under a 60X oil objective lens with a Nikon A1RS confocal microscope. Immuno-fluorescence from central and mid-peripheral regions was quantified using ImageJ.

### Proximity ligation assay to detect RIP1/RIP3 interaction

Proximity ligation assay was performed following our published method [29], using the Duolink® In Situ Red Starter Kit (Mouse/ Rabbit), which was purchased from Sigma-Aldrich (DUO92101). Cryosections were blocked with 5% normal donkey serum in PBS containing 0.1 Triton-X 100 for 1 h at room temperature. Mouse anti-RIP1 (BD Transduction Laboratories, 610,459, 1:100) and Rabbit anti-RIP3 (ProSci, 2283, 1:100) were then incubated with the sections overnight. PLA® probes incubation, ligation, amplification, and mounting followed manufacturer’s instructions. Images were taken and analyzed using a Nikon A1RS confocal microscope.

### Tyramide signal amplification (TSA)-enhanced immunostaining to detect cleaved-Caspase3

TSA enhanced immunostaining has been described previously [30]. Briefly, retinal sections were first incubated with 1% H_2_O_2_ for 1 h at room temperature to quench endogenous peroxidase, then blocked with 5% BSA, 0.1% Triton-X 100 in PBS for another hour at room temperature. Following incubation with rabbit anti-ACTIVE® Caspase-3 pAb (Promega, G7481, 1:250) and HRP-conjugated goat anti-rabbit secondary antibody (Jackson ImmunoReseach, 115–035-003, 1:1000), the sections were washed twice, 3 min each, in 100 mM borate (pH 8.5) supplemented with 0.1% Tween-20. The sections were then incubated with Tyramide-CF568 (Biotium, 92,173, 1 μg/ml) in TSA reaction buffer (100 mM borate, pH 8.5, 0.1% Tween-20, 0.003% H_2_O_2_) for 30 min at room temperature. After washing with PBS + 0.1% Tween-20, the sections were counterstained with Hoechst 33,342 prior to imaging with a Nikon A1RS confocal microscope.

### Western blotting for assessment of protein levels

To compare protein levels between experimental conditions, two retinas were collected from one mouse of each condition, homogenized and then subjected to Western blotting. Thus, each lane on the blot represents two retinas of the same mouse of one experimental condition. The retina homogenization/Western blotting experiment was repeated 3 or 4 times each time with a different mouse of the same experimental condition (one lane, one condition). Normalized values (of the same condition) from 3 or 4 blots were averaged.

Retinal homogenates were prepared [27] and solubilized in RIPA buffer containing protease inhibitors (50 mM Tris, 150 mM NaCl, 1% Nonidet P-40, 0.1% sodium dodecyl sulfate, and 10 μg/ml aprotinin). Protein concentrations of cell lysates were determined using a Bio-Rad DC™ Protein Assay kit. Approximately 15–30 μg of proteins from each sample was separated on 4–20% Mini-PROTEAN TGX precast gels (Bio-Rad) and transferred to PVDF membranes. Sources and dilutions of primary antibodies are the following: Mouse anti-RIP1 (BD Transduction Laboratories, 610,459), 1:1000, Rabbit anti-RIP3 (ProSci, 2283), 1:1000, Cleaved Caspase3 (Asp175) (5A1E) Rabbit mAb (Cell Signaling, 9664), 1:1000, Rabbit anti-IBA-1 (Novus, NBP2–19019), 1:1000; Rabbit anti-GFAP (Cell Signaling, 12,389), 1:1000; rabbit anti-CHOP (Santa Cruz, sc8327), 1:100; Rabbit anti-LC3 (Sigma-Aldrich, L7543), 1:5000, and mouse anti-β-actin (Sigma-Aldrich, A2228), 1:10,000. After incubation with HRP-conjugated secondary antibodies (1:5000, goat anti-rabbit or mouse, Bio-Rad), specific protein bands on the blots were visualized by applying enhanced chemiluminescence reagents according to the manufacturer’s instructions (Pierce, Rockford, IL) and then recorded with a LAS-4000 Mini imager (GE, Piscataway, NJ). Band intensity was quantified using ImageJ.

### Electroretinogram recording for rod photoreceptor function

ISCEV standard full-field flash ERG was performed using HMsERG system (OcuScience, Henderson, NV) following our published method [31]. Mice were dark-adapted overnight and anesthetized with intraperitoneal ketamine (90 mg/kg) and xylazine (8 mg/kg) under dim-red illumination. After topical application of tropicamide (1%, Alcon) and phenylephrine (2.5%, Alcon) for pupillary dilation and proparacaine hydrochloride (0.5%, Alcon) for topical anesthesia, stainless steel subdermal needle electrodes were placed for ground (at the tail) and under individual eye lids as reference electrodes. Rodent 2.5 mm contact lens with silver-embedded thread electrode were placed on cornea of each eye using Goniovisc hypromellose 2.5% ophthalmic lubricant solution (HUB Pharmaceuticals, CA). Flash ERG recordings were obtained simultaneously from both eyes at increasing light intensities from 0.03 to 30 cd⋅s/m^2^ (saturating intensity in our reported studies [31]) under dark-adapted conditions. The stimulus interval between flashes varied from 20 s at the lowest stimulus strengths to 60 s at the highest ones. Two to 10 responses were averaged depending on flash intensity. ERG signals were sampled at 1 kHz and recorded with 0.3 Hz low-frequency and 300 Hz high-frequency cutoffs. Analysis of a-wave and b-wave amplitudes was performed using ERGView analytical software (OcuScience, Henderson, NV) that digitally filters out high-frequency oscillatory potential wavelets. The a-wave amplitude was measured from the baseline to the negative peak, and the b-wave was measured from the a-wave trough to the maximum positive peak and plotted using Origin.

### Morphometric analyses of photoreceptor degeneration and ganglion cell numbers

Retinal cryosections were Hoechst-stained for counting photoreceptor numbers with two approaches in parallel, following our published method with minor modifications [20, 27]. Briefly, on each sagittal section, the regions of 0–1000 μm and 1000–2000 μm from the optic nerve head were designated as central and mid-peripheral, respectively. Four fields were chosen in the central and mid-peripheral regions of the outer nuclear layer flanking the optic nerve head. In each of the four fields, the ONL thickness (first approach) or nuclei number (second approach) were manually measured. The values from all 3–4 sections of the same animal were averaged, and the means from 3 to 4 animals were then averaged to calculate the mean and standard error (SE) for each group of animals. Using the same method, we counted and quantified the numbers of both RGCs (BRN3A-positive) and total cells (nuclei in the RGC layer).

### Statistical analysis

For morphometric analyses, fluorescence micrographs of Hoechst-stained retinal sections, selection of fields in ONL or GCL, and ONL thickness measurement and nuclei counting were all performed by students blinded to experimental grouping information. All data are presented as mean ± SE (standard error) of at least three independent experiments. Statistical significance (set at *P* < 0.05) was assessed by two-tailed unpaired Student’s-t-test performed with GraphPad Prism.

## Results

### Cone PR degeneration is exacerbated due to S1R knockout in rd10 mice

**Fig. 1:**
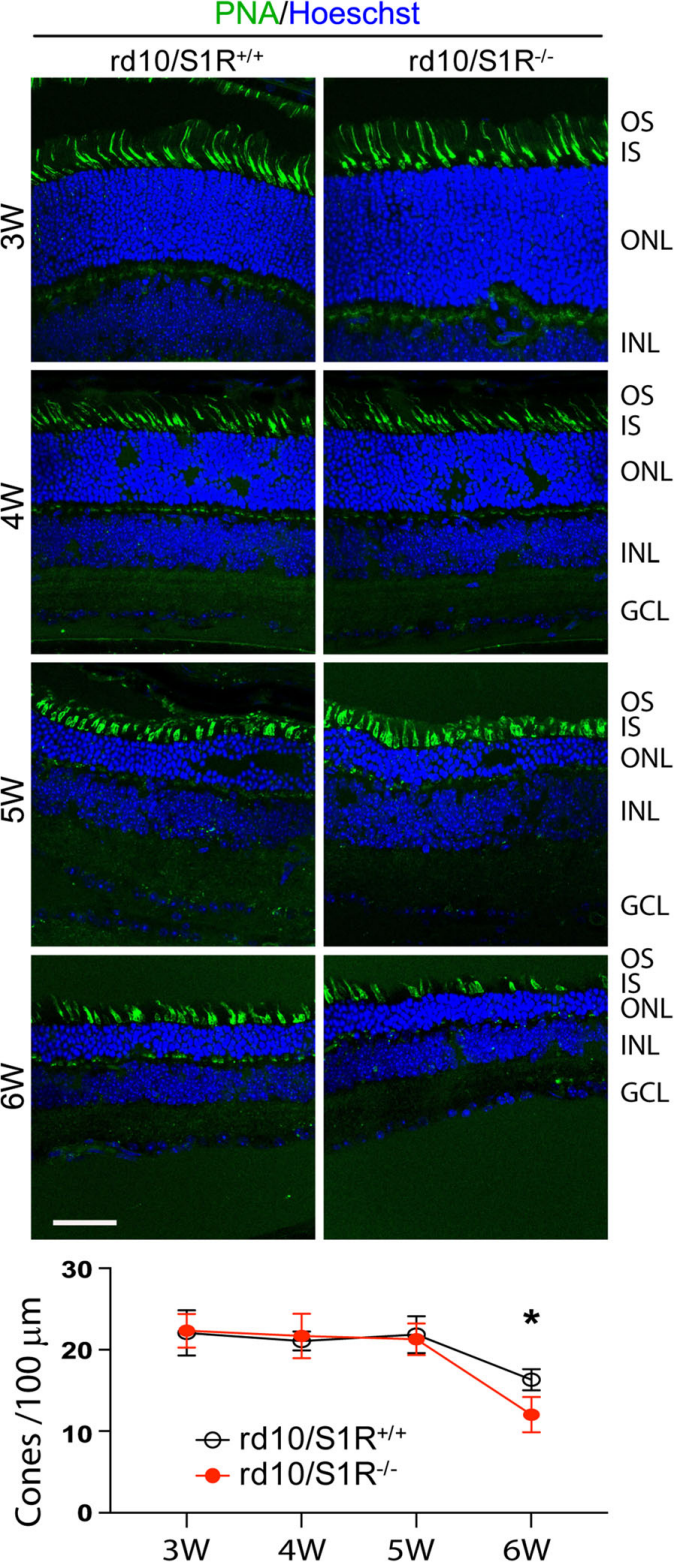
PNA label indicating exacerbated cone loss due to S1R knockout in rd10 mice. Animals were euthanized at indicated time points, and retinal sections were prepared for cone labeling with fluorescent PNA. OS/IS: outer/inner segment; ONL: outer nuclear layer; INL: inner nuclear layer; GCL: ganglion cell layer. Scale bar*:* 20 μm. Quantification: cone cell number per 100 μm ONL length; mean ± SE, *n* = 3–4 mice; *P < 0.05

In order to determine S1R-specific cellular and molecular responses during PR degeneration, we used a crossed line of rd10/S1R^−/−^ to compare with rd10 mice (rd10/S1R^+/+^) throughout this study. The rd10 retina undergoes rapid PR degeneration [23], rendering time-dependent mechanistic study difficult. To circumvent this problem, we reared mice in a dedicated room of dim red light (<5 lx, 12 h/12 h light/dark) to slow down PR death. Cone PR degeneration was compared between rd10/S1R^−/−^ and rd10/S1R^+/+^ mice via PNA labeling on retinal sections (Fig. 1). The data show that cone loss in rd10 mice (with or without S1R) did not occur until postnatal 6 weeks (6 W), indicating slower PR degeneration compared to rd10 mice reared in regular light [24, 27]. This result also informs that in our experimental setting PR loss before 5 W occurred predominantly in rods. Furthermore, the comparison between the rd10 mice with and without S1R shows that cone loss was significantly accelerated at 6 W in the absence of S1R. This result is consistent with the observation by Wang et al. that activation of S1R in rd10 mice with (+)-pentazocine rescues cones [23].

### Activation of the RIP1/RIP3 pathway in rd10 retinas is accelerated due to S1R knockout

**Fig. 2:**
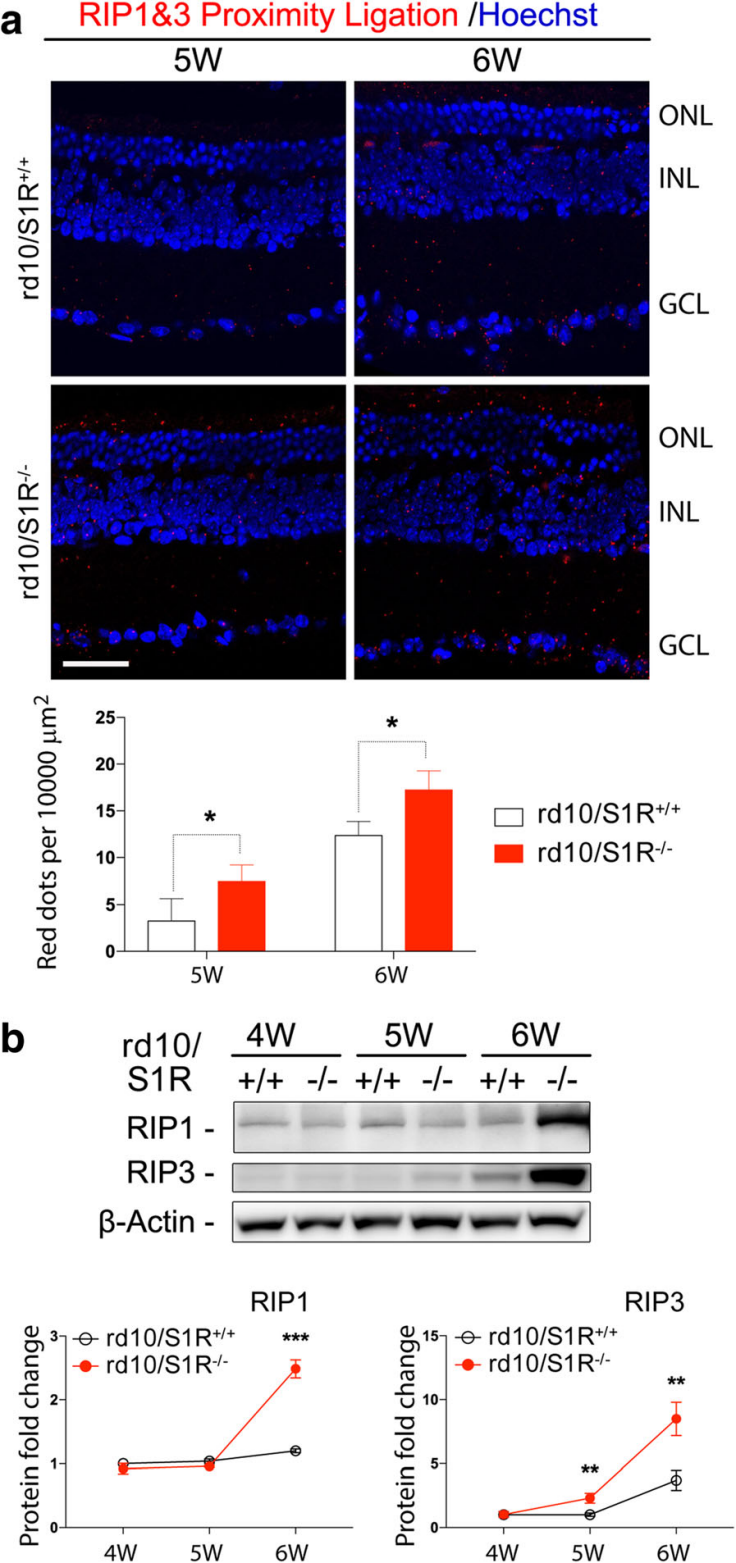
Up-regulation of RIP1 and RIP3 proteins and their interaction (proximity ligation) in rd10 retinas due to S1R knockout. Animals were euthanized at indicated time points, and retinal sections or homogenates were prepared for RIP1/RIP3 proximity ligation assay and Western blotting, respectively. **a**. Proximity ligation. ONL: outer nuclear layer; INL: inner nuclear layer; GCL: ganglion cell layer. Scale bar*:* 20 μm. Quantification: mean ± SE, *n* = 3–4 mice; *P < 0.05. **b**. Quantification of Western blots. Shown is one of four blots each representing an independent experiment. Each lane represents one mouse, from which two retinas were homogenized together and subjected to Western blotting. Normalized (to beta-actin) values of the same condition from four experiments (blots) were averaged to calculate mean ± SE; *n* = 4 mice; **P < 0.01, ***P < 0.001

A recent study discovered that whereas apoptosis occurs early when rod degeneration was prevailing, RIP3-mediated necroptosis was responsible for the death of cones while apoptosis was diminishing [25]. Whether S1R influences necroptosis in rd10 cones was not known. We used an approach of proximity ligation [29] to assess RIP1/RIP3 interaction, an essential step leading to the activation of necroptosis [25]. Excellent specificity of this assay was manifested by the negative control (Additional file 1). As cone loss started after 5 W under our experimental conditions (Fig. 1), we determined RIP1/RIP3 interaction at the 5 W and 6 W time points (Fig. 2a). While a small number of ligation-positive dots were observed at 5 W in rd10/S1R^+/+^ retinas, the number increased by ~3 fold in rd10/S1R^−/−^ retinas, indicating accelerated RIP1/RIP3 interaction in the absence of S1R. At 6 W, many more ligation-positive dots appeared in rd10/S1R^+/+^ retinas, consistent with cone death at 6 W revealed in Fig. 1, and the number was significantly higher in rd10/S1R^−/−^ retinas. We then performed Western blotting to confirm this result. As shown in Fig. 2b, in contrast to a slight increase of RIP1 and RIP3 proteins after 5 W in rd10/S1R^+/+^ retinas, these two proteins were dramatically up-regulated in rd10/S1R^−/−^ retinas. It is also worth noting that while the RIP3 protein level in rd10/S1R^+/+^ retinas remained stable at 5 W relative to 4 W, it already rose to a 2-fold higher level in rd10/S1R^−/−^ retinas at 5 W. These results reveal that the RIP1/RIP3 interaction was accelerated and enhanced in rd10 retinas without S1R.

### Caspase3 activation in rd10 PR cells is accelerated due to S1R knockout

**Fig. 3:**
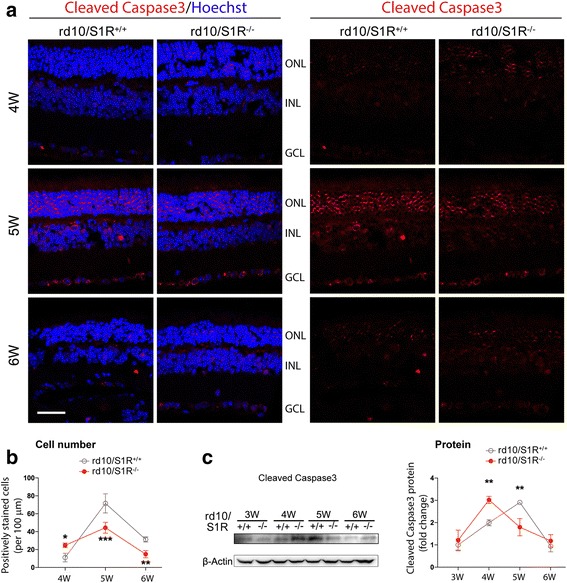
Accelerated production of cleaved Caspase3 in rd10 retinas due to S1R knockout. **a**. TSA-enhanced immunostaining of cleaved Caspase3 on retinal sections collected at indicated time points. The one-color (channel) version is shown on the right. ONL: outer nuclear layer; INL: inner nuclear layer; GCL: ganglion cell layer. Scale bar*:* 20 μm. **b**. Quantification of cells stained positively for cleaved Caspase3. Mean ± SE, *n* = 3–4 mice; *P < 0.05, **P < 0.01, ***P < 0.001. **c**. Quantification of Western blots showing cleaved Caspase3 in retinal homogenates. For the method of quantification refer to Fig. 2b; mean ± SE; *n* = 4; **P < 0.01

To investigate S1R’s influence on apoptosis, the mode of rod death [25], we used a TSA-enhanced method for immunostaining of cleaved Caspase3, an indicator of Caspase3-mediated apoptotic activation. As shown in Fig. 3a, the nuclei stained positive for cleaved Caspase3 predominantly localized in ONL with a small number in the inner layers. In rd10/S1R^+/+^ mouse retinas, positively stained cells peaked at 5 W and then diminished at 6 W (Fig. 3b). In rd10/S1R^−/−^ retinas, the number of positive cells was significantly higher than that in rd10/S1R^+/+^ retinas at 4 W but lower at later time points. We then assessed cleaved Caspase3 protein level changes in retinal homogenates collected throughout 3 W–6 W (Fig. 3c). Consistent with the immunostaining data, whereas cleaved Caspase3 in rd10/S1R^+/+^ retinas peaked at 5 W, its highest level in rd10/S1R^−/−^ retinas was observed at 4 W. These results indicate that S1R knockout in rd10 mice accelerated Caspase3 activation.

### Müller glial but not microglial activation in rd10 retinas is accelerated due to S1R knockout

**Fig. 4:**
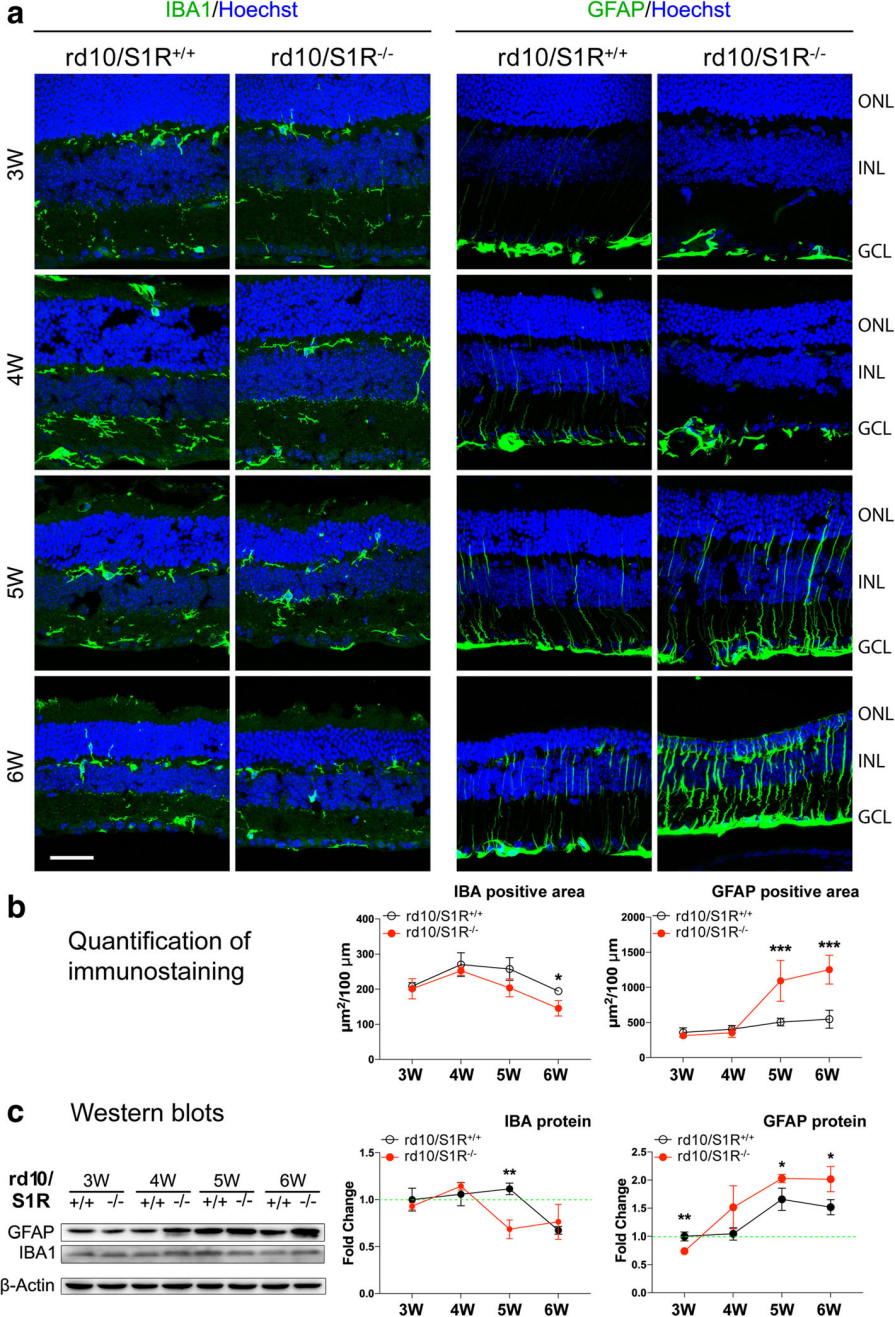
Enhanced Müller glial activation in rd10 retinas due to S1R knockout. **a**. Immunostaining of IBA1 and GFAP on retinal sections collected at indicated time points. ONL: outer nuclear layer; INL: inner nuclear layer; GCL: ganglion cell layer. Müller cells are distinguishable by their radial morphology. Scale bar*:* 20 μm. **b**. Quantification of immunostained cell area to assess increase of glial activation. Mean ± SE, *n* = 3–4 mice, *P < 0.05, ***P < 0.001. **c**. Quantification of Western blots (shown on the *left*) detecting IBA1 and GFAP in retinal homogenates. For the method of quantification refer to Fig. 2b; mean ± SE; *n* = 3–4; *P < 0.05, **P < 0.01

Gliosis, or activation of microglia and macroglia (Müller cells and astrocytes) has been reported to contribute to PR death in rd10 mice [24]. Moreover, it was recently shown that S1R activity influences activation of microglia and Müller cells of the retina [32, 33]. We thus performed immunostaining (Fig. 4a) and Western blotting of IBA1 and GFAP, commonly used markers for these retinal glial cells, respectively [24]. Quantification of immunostaining shows distinct time-dependent changes of IBA1 and GFAP (Fig. 4b). In rd10/S1R^+/+^ retinas, while IBA1 staining increased from 3 W to 4 W and then decreased to the 3 W level, there was a slight increase of GFAP staining after 4 W. In rd10/S1R^−/−^ retinas, the pattern of IBA1 change was very similar to that in rd10/S1R^+/+^ retinas albeit with slightly lower levels at 5 W and 6 W. In stark contrast, GFAP rapidly increased after 4 W in rd10/S1R^−/−^ retinas, and was more than twofold higher than in rd10/S1R^+/+^ retinas at 5 W and 6 W. In good agreement with the immunostaining data, Western blots (Fig. 4c) show similar patterns of IBA1 protein time course in rd10 retinas with and without S1R, although at 5 W IBA1 was significantly lower in the absence of S1R. Whereas in rd10/S1R^+/+^ retinas GFAP protein did not increase until 5 W, in rd10/S1R^−/−^ retinas up-regulation of GFAP started by 4 W, indicating accelerated macroglia activation. Moreover, rd10/S1R^−/−^ GFAP protein levels became significantly higher than the rd10/S1R^+/+^ control at 5 W and 6 W. It is also interesting to note that opposite to later time points, at 3 W GFAP was lower in rd10/S1R^−/−^ retinas than in the rd10/S1R^+/+^ control. Together, these results indicate that activation of macroglia but not microglia strongly responded to the absence of S1R in rd10 retinas. The radial staining (Fig. 4a) suggests that Müller cells were responsible for the observed macroglia activation.

### Biphasic changes of ER stress response and autophagy in rd10 retinas in response to S1R knockout

**Fig. 5:**
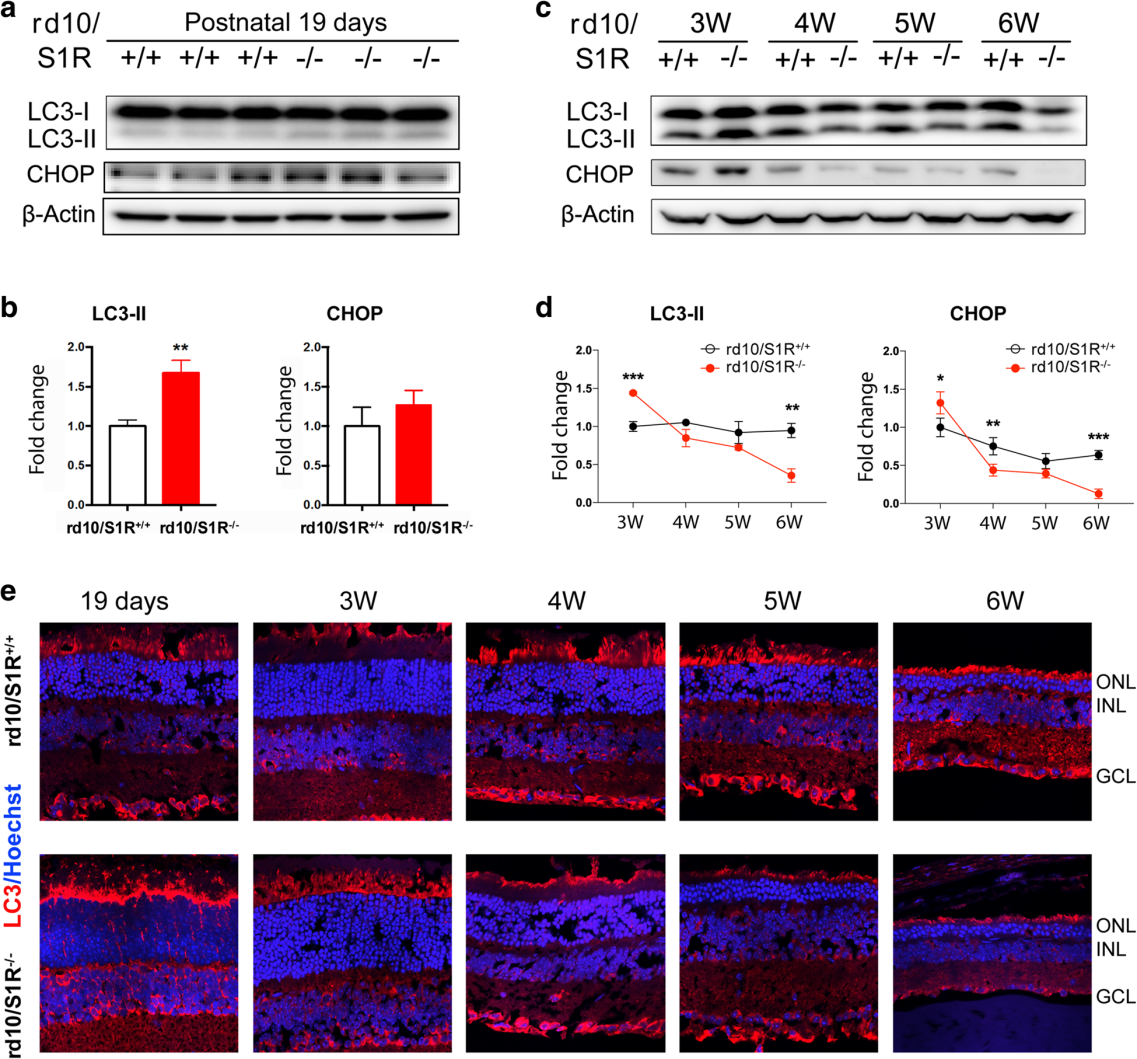
Changes of LC3-II and CHOP protein levels during PR degeneration in rd10 retinas with or without S1R. **a** and **c**. Western blots detecting LC3-I (uncleaved), LC3-II (cleaved and lipidated, marker of autophagosomes), and CHOP in retinal homogenates collected at indicated time points. The postnatal day-19 experiment (see **a**) was performed about 6 months after that in **c**, triplicate samples (each from two retinas in one mouse) were used for the same blot. **b** and **d**. Quantification of Western blots shown in **a** and **c**, respectively. For the method of quantification refer to Fig. 2b; mean ± SE; *n* = 4 mice; *P < 0.05, **P < 0.01, ***P < 0.001. **e**. Immunostaining of LC3 on retinal sections collected at indicated time points

Up to this point, our results showed an overall protective role of S1R in rd10 retinas in a later phase (4 W–6 W). We thus further explored a possible S1R influence on cytoprotective mechanisms in rd10 retinas. In previous studies, S1R has been closely linked to autophagy [34, 35] and ER stress response [1, 36], two of the most prominent cytoprotective processes. We determined the effects of S1R knockout on autophagy and ER stress response, via Western blot analysis of their respective protein markers, LC3-II and CHOP (Fig. 5, a-c). As shown in Fig. 5d, time-dependent changes of these two markers exhibit similar patterns. Both LC3-II and CHOP remained largely stable from 3 W to 6 W in rd10/S1R^+/+^ retinas. In contrast, in rd10/S1R^−/−^ retinas these two markers showed highest levels at 3 W and then continuously decreased and finally diminished at 6 W; they became lower than those in rd10/S1R^+/+^ retinas after 4 W. It is interesting to note that at 3 W both LC3-II and CHOP protein levels were higher in rd10/S1R^−/−^ retinas than in the rd10/S1R^+/+^ control. This result was further confirmed at an additional early time point of postnatal day 19 (Fig. 5, a and b). Furthermore, LC3-II immunostaining on retinal sections collected at time points from day 19 to 6 W (Fig. 5e) showed the same trend of the Western blot data. Overall, the results obtained here revealed biphasic responses of LC3-II and CHOP to S1R knockout in rd10 retinas: first up-regulation (day 19 and 3 W) and then down-regulation (4 W–6 W).

### Biphasic effect of S1R knockout in rd10 mice on rod PR electrophysiology

**Fig. 6:**
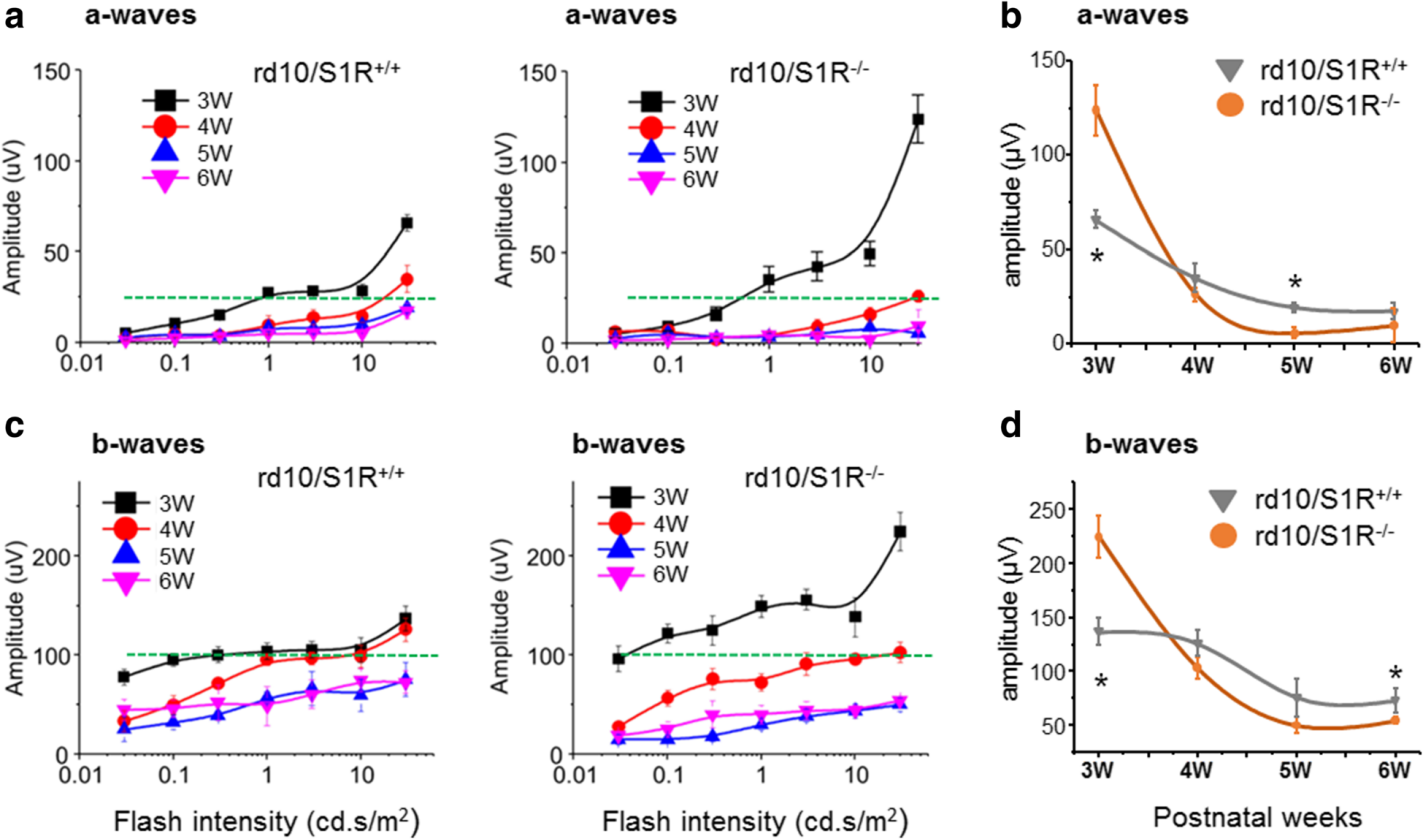
Effect of S1R knockout on rod electrophysiological responses in rd10 mice. Rod ERG recording was performed with scotopic flash stimulation at indicated flash intensities and time points, as described in Methods. **a**. Average plot of a-wave amplitudes of rd10/S1R^+/+^ mice and rd10/S1R^-/-^ mice. The dashed green line serves as a reference for comparison between two plots. **b**. Comparison of a-wave amplitude averages (30 cd.s/m^2^) in the presence and absence of S1R. **c**. Separately plotted average b-wave amplitudes of rd10/S1R^+/+^ and rd10/S1R^-/-^ mice. **d**. Comparison of b-wave amplitude averages (30 cd.s/m^2^) in the presence and absence of S1R. ERG a-wave is the downward deflected negative response; b-wave is from the a-wave peak to positive response peak. Quantification: Mean ± SE, *n* = 5–7 mice; *P < 0.05

The foregoing opposite outcomes at early and late time points prompted us to determine a 3 W–6 W “full-spectrum” impact of S1R knockout on rods, considering that rod degeneration occurs earlier and consequently leads to cone death [25]. We thus determined scotopic light-stimulated ERG, the a-waves derived from which reflect the function of rod photo-transduction [31]. As indicated by the average data in Fig. 6a, at the 3 W time point, a-wave amplitudes were markedly higher in rd10/S1R^−/−^ (Fig. 6a, right panel) versus rd10/S1R^+/+^ mice (Fig. 6a, left panel) at flash intensities greater than 1.0 cd.s/m^2^, suggesting a protective effect of S1R knockout. In stark contrast, at later time points (4 W–6 W) the opposite occurred as measured at 30 cd.s/m^2^ (Fig. 6b). At lower flash intensities the difference between rd10/S1R^−/−^ and rd10/S1R^+/+^ was smaller. Following a similar pattern, b-wave amplitudes were higher at 3 W but lower at later time points in rd10/S1R^−/−^ mice than in rd10/S1R^+/+^ mice (Fig. 6, c and d). While b-wave is indicative of inner retina neurons especially bipolar cells, they are strongly influenced by the rod PR function. Combined, the ERG recordings demonstrate a biphasic effect of S1R knockout in rd10 mice on the rod photoresponse, i.e., augmentation at 3 W, and then attenuation at later time points.

### Biphasic effect of S1R knockout in rd10 mice on photoreceptor degeneration

**Fig. 7:**
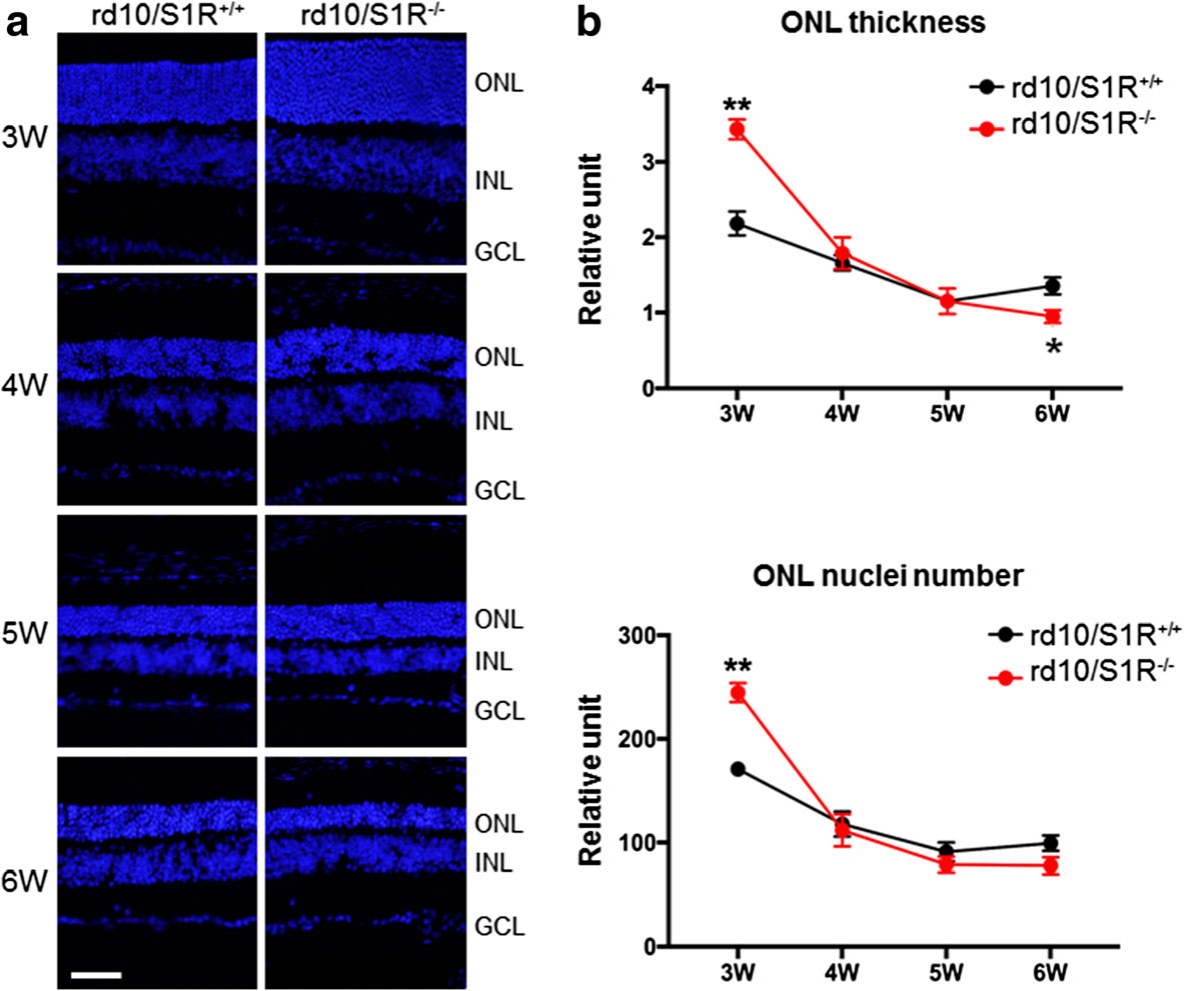
Morphometric analysis of the effect of S1R knockout on PR survival in rd10 mice. **a**. Hoechst staining of nuclei on retinal sections collected at indicated time points. ONL: outer nuclear layer; INL: inner nuclear layer; GCL: ganglion cell layer. Scale bar*:* 20 μm. **b**. Quantification of ONL thickness and nuclei numbers: Mean ± SE, *n* = 3–4 mice; *P < 0.05, **P < 0.01

To further confirm this seemingly paradoxical biphasic effect of S1R knockout on PR cells, we performed histological evaluation by two approaches: measuring ONL thickness and counting nuclei (Fig. 7a). As shown in Fig. 7b, these two methods produced the same result, which agrees well with the ERG data. PR loss occurred mainly between 3 W to 5 W, and it was exacerbated in rd10/S1R^−/−^ retinas compared to rd10/S1R^+/+^ retinas at 6 W, consistent with the cone PNA staining data (Fig. 1). Moreover, confirming the ERG results, at 3 W both the ONL thickness and nuclei number were markedly higher in rd10/S1R^−/−^ versus rd10/S1R^+/+^ retinas (Fig. 7b). Since cone loss did not occur until after 5 W (Fig. 1), it is safe to infer that it was rods that showed changes in ONL at 3 W.

**Fig. 8:**
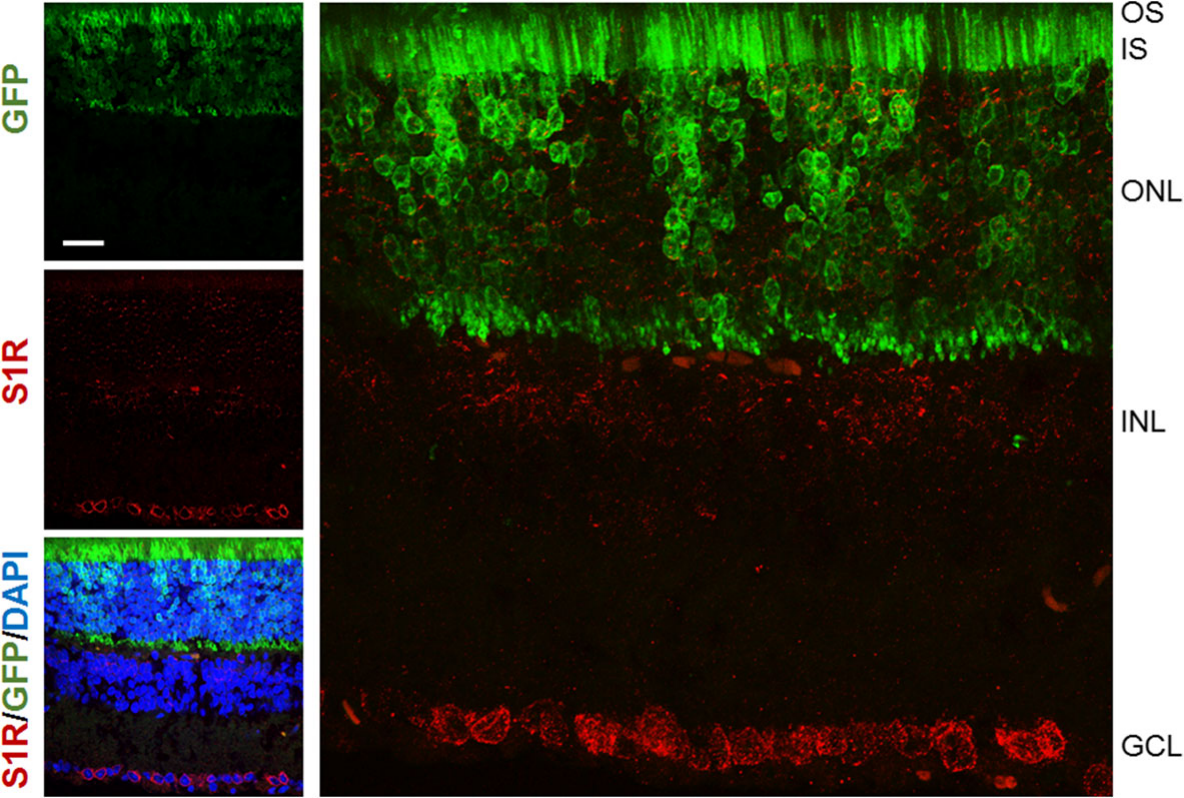
Immunostaining of S1R showing its presence in rods. Adult Nrl-GFP mouse retinal sections were prepared for immunostaining of S1R using an in-house produced antibody that previously proved highly specific [21, 28, 42]. GFP expressed with an Nrl promoter illuminates rods. An enlarged picture on the *righ*t shows GFP-labeled rods and S1R-positive staining. Scale bar*:* 20 μm

In view of the profound S1R influence on rod survival and function, a follow-up question is whether S1R actually expresses in rods. Although previous studies detected the S1R protein in ONL [21, 28], its distribution has never been distinguished between rods and cones. Here we immunostained S1R on retinal sections from a transgenic mouse line expressing GFP in an Nrl-driven, rod-specific manner. As indicated in the ONL of the fluorescent image (Fig. 8), while S1R-positive staining was found in GFP-positive rod cells, it also localized in GFP-negative cells, which most likely were cones because there are essentially only rod and cone photoreceptor nuclei in ONL.

### S1R knockout increases RGCs at 3 W in rd10 retinas

**Fig. 9:**
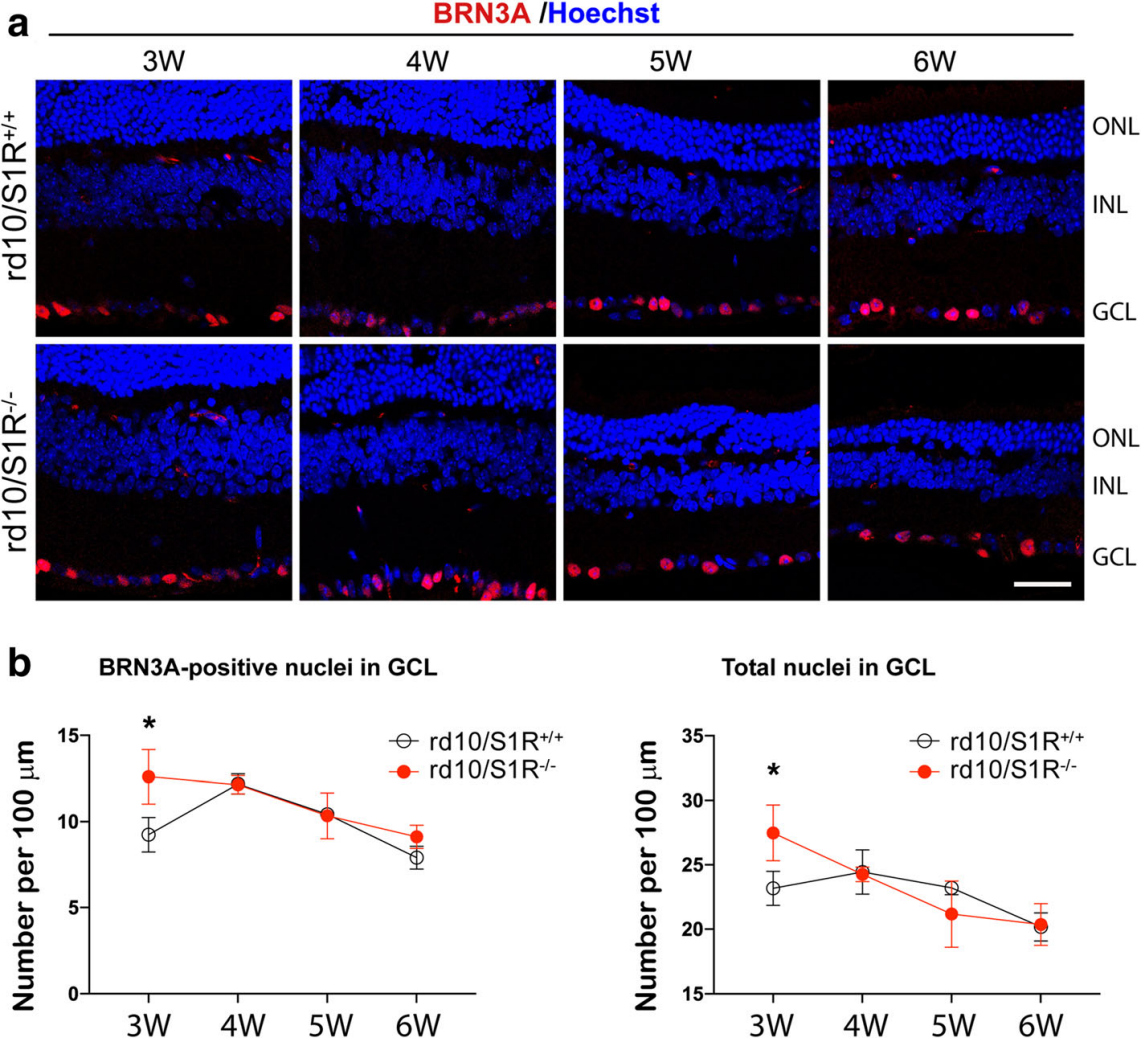
Effect of S1R knockout on RGC numbers in rd10 mice. **a**. Immunostaining of BRN3A on retinal sections collected at indicated time points. ONL: outer nuclear layer; INL: inner nuclear layer; GCL: ganglion cell layer. Scale bar*:* 20 μm. **b**. Quantification: Mean ± SE; *n* = 3–4 mice; *P < 0.05

It is worth noting that cells detected positive for cleaved Caspase3 and RIP1/RIP3 ligation were also observed in inner retinal layers (INL and GCL) at 5 W and 6 W (Figs. 2 and 3). This implicates possible loss of secondary neurons, in particular RGCs, another type of retinal neurons critical to vision but vulnerable to stress-induced degeneration. We thus compared the numbers of RGCs between rd10/S1R^−/−^ and rd10/S1R^+/+^ mice by immunostaining BRN3A, a commonly used RGC-specific marker [20] (Fig. 9a). Our data show that while the RGC number slowly decreased, it was not affected by S1R knockout, except at 3 W (Fig. 9b). Interestingly, at this early time point, the number of BRN3A-positive RGCs was significantly higher in rd10/S1R^−/−^ retinas compared to rd10/S1R^+/+^ retinas.

## Discussion

RP is the most common group of inherited retinal degenerations affecting one in 3500–5000 individuals, but there are no effective treatments available [24]. The S1R chaperone emerges as a potential intervention target shared in different types of retinal degenerations [19, 20, 22, 23]. Of particular interest, Wang et al. very recently reported that pharmacological activation of S1R rescues cones in the rd10 model of RP that is characteristic of sequential death of rods and cones [23]. In our study, by comparing dim light-reared rd10/S1R^−/−^ versus rd10/S1R^+/+^ mice during a time course spanning early and late stages of PR degeneration, we were able to determine “time-resolved” specific roles of S1R in rod and cone degenerations. We made two major new findings: 1) RIP-indicated activation of necroptosis, the recently identified chief mechanism of rd10 cone death [25], was amplified due to S1R knockout; 2) likely contributing to cone death, rod degeneration was exacerbated at later stages (4 W–6 W) due to S1R knockout, although paradoxically alleviated at an earlier stage (3 W). We further identified that autophagy and ER stress response may at least partially account for these outcomes.

The rd10 model first undergoes autonomous rod degeneration and then cone death triggered by dying rods [25]. While Caspase-mediated apoptosis is known to be the major mode of rod death, mechanisms underlying cone death are less well understood. Recently, Murakami et al. demonstrated that RIP3-mediated necroptosis is the mechanism dominating cone death in rd10 mice yet with little impact on rods [25]. Thus, apoptosis and necroptosis, two distinct modes of cell death, account for sequential loss of rd10 rods and cones, respectively [25]. In a good agreement with their report, our data showed that in rd10 mice (rd10/S1R^+/+^), cleaved Caspase3 peaked at 5 W and diminished at 6 W whereas levels of RIP1/RIP3 proteins and their interaction did not rise until 6 W, the time point when cone loss was observed (Fig. 1). Inasmuch as cone loss adversely impacts daily living, Wang et al. made a very significant finding that injecting (+)-pentazocine to activate S1R markedly preserved cones in rd10 mice [23]. Whether this protection of cones involves a S1R-specific effect on the RIP pathway of necroptosis remained unknown. Here we found that compared to rd10/S1R^+/+^ mice, increases of retinal RIP1 and RIP3 proteins as well as their interaction occurred earlier (at 5 W) and were substantially enhanced in rd10/S1R^−/−^ mice. Considering that cone loss occurred later (at 6 W), these results suggest that intensified necroptosis activation in the absence of S1R is an important contributor to aggravated cone death.

S1R influences necroptosis activation possibly by multiple mechanisms. First, it is known that glial release of inflammatory cytokines (e.g., TNFα) and reactive oxygen species (ROS), a hallmark of reactive gliosis [24, 27], potently stimulates necroptosis in the retina [37, 38]. Consistently, our data show that in rd10 retinas without (versus with) S1R, GFAP-indicated macroglial activation was substantially elevated. Although both astrocytes and Müller cells express GFAP [24], it was the characteristic Müller cell radial morphology that showed a burst at 5 W and 6 W in rd10/S1R^−/−^ retinas (Fig. 4, a and b), accompanied by a marked up-regulation of RIP1/RIP3 expression and interaction. It is worth noting that in our experimental setting, we did not observe a major difference in IBA1-indicated microglial activation between the rd10 retinas without and with S1R, and IBA1 expression remained largely stable during the 3 W–6 W time course. Taken together, Müller glia appear to be the main contributor to intensified gliosis and necroptosis activation in rd10/S1R^−/−^ retinas. An alternative explanation might be that necroptotic cones and ROS production thereof triggered Müller cell activation. However, a surge of GFAP occurred ahead (at 5 W) of RIPs (at 6 W) in rd10/S1R^−/−^ retinas, arguing for a probable sequence of Müller cell gliosis and then necroptosis stimulation. Second, the two types of S1R-associated stress-response activities [1, 34, 36], indicated by LC3-II and CHOP, declined over time in the absence of S1R and diminished at 6 W. Elimination of these activities is expected to result in elevation of necroptosis stimulants such as inflammatory cytokines and ROS. Third, S1R may directly regulate necroptosis in cones independent of reactive gliosis, although at present little evidence is available. Elucidation of this scenario, while beyond the scope of the current study, requires future experiments of conditional S1R knockout in rd10 cones. Fourth, given that in rd10 mice cones die as a secondary event triggered by dying rods [25], logically, aggravated rod death, for instance due to S1R knockout, should consequently exacerbate cone loss.

Therefore, how S1R influences rod degeneration in rd10 mice is an important question. It remained unclear likely because the previous study primarily focused on the peak time of cone death, at which point rod loss was almost complete [23]. In our experiments using mice reared under dim light, good levels of a-waves and b-waves were preserved at 3 W and 4 W and residual signals were still detectable at 5 W and 6 W in rd10/S1R^+/+^ mice. In comparison, a-wave and b-wave amplitudes were reduced at 4 W–6 W in rd10/S1R^−/−^ mice (Fig. 6, b and d). Consistently, ONL thickness and nuclei numbers followed the same trend. Thus, our data support a protective role of S1R for rods in rd10 retinas during 4 W–6 W. Further supporting this assertion, S1R knockout in rd10 mice accelerated Caspase3-associated apoptosis, the known chief mechanism of rod death. In addition, in the Wang et al. report [23], injection of S1R agonist (+)-pentazocine started at 2 W and continued to 6 W, spanning the course of rod degeneration, and may have reduced cone death by ameliorating rod degeneration. Moreover, tracing rods by Nrl-driven GFP expression in the mouse retina, we verified the presence of S1R in rods via S1R immunopositivity.

**Fig. 10:**
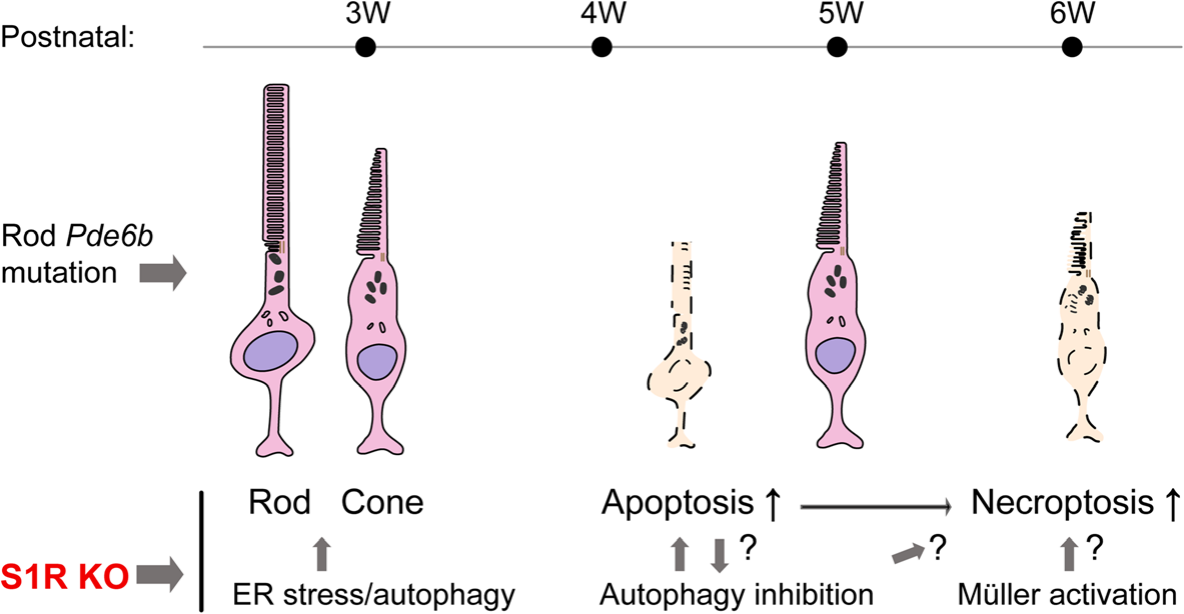
Schematic of the impact of S1R knockout on rd10 mouse retinal degeneration. The time course shows autonomous rod degeneration featuring apoptosis initiated by a mutation in the rod photoreceptor Pde6b gene, followed by secondary cone death with necroptosis as the major mechanism. S1R knockout in the rd10 mouse retina enhances ER stress and autophagy at early stage but leads to autophagy inhibition and Müller glial activation at later time points, which are accompanied by exaggerated rod and cone death

An unexpected finding in this study is the seemingly paradoxical rod protection at 3 W due to S1R knockout in rd10 mice. A robust preservation of rods at 3 W due to S1R knockout is evidenced not only by the statistics of ONL thickness and nuclei numbers but also rod ERG recordings. Thus, the role of S1R during the 3 W–6 W full time course appears to be biphasic. Interestingly, biphasic profiles were also observed with LC3-II and CHOP. Whereas levels of these two proteins were higher in rd10/S1R^−/−^ versus rd10/S1R^+/+^ retinas at 3 W, the opposite occurred at 4 W–6 W. Autophagy is a critical cytoprotective mechanism in the rd10 retina [39]. ER stress response pathways including PERK, IRE1, and ATF6 are also generally cytoprotective [36]; CHOP is a hub effector downstream of these pathways and often associated with ER stress-mediated apoptosis [40, 41]. Increasing evidence indicates S1R participation in autophagy [34, 35] and ER stress response under stress conditions [1, 36]. On the other hand, compromised S1R (an ER chaperone) per se is an ER stress signal that may activate ER stress responses and autophagy in cells with low basal stress. For example, elevated LC3-II or autophagy upon S1R inhibition has been reported in non-retinal tissues [35], although whether S1R influences autophagy in retinas has not been documented. Based on previous reports and our own data, we put forward here a reconciliative explanation for the biphasic role of S1R in rd10 retinas (Fig. 10). When cell stress is minor at the onset of rod degeneration (~3 W), the absence of the S1R is an ER stress signal activating autophagy and ER stress response, which protect rods from dying. As insults in rods become severe, apoptosis is activated (e.g., by elevated Ca^2+^) [39] and cytoprotective pathway (LC3-II) is inhibited [39]. This is particularly evident in the absence of S1R’s protective actions, as shown by more rod (and subsequently cone) death in rd10/S1R^−/−^ than in rd10/S1R^+/+^ retinas.

The slow degeneration of RGCs in rd10 retinas observed here likely resulted from collateral damage inflicted by dying PR cells, as evidenced by detections positive for cleaved Caspase3 and RIP1/RIP3 ligation. The lack of difference in RGC numbers between rd10/S1R^−/−^ and rd10/S1R^+/+^ retinas also informs that RGC death is secondary to the PR degeneration. Interestingly, a greater RGC number in rd10/S1R^−/−^ versus rd10/S1R^+/+^ retinas was observed at 3 W, seemingly indicative of RGC protection by S1R knockout. However, unlike rods, from 3 W to 4 W RGCs did not degenerate but rather increased in rd10/S1R^+/+^ retinas. This suggests that a higher number of RGCs at 3 W in rd10/S1R^−/−^ retinas was not because of faster RGC degeneration in rd10/S1R^+/+^ retinas. Understanding the exact underlying mechanism awaits future detailed investigation. Nonetheless, the results of RGCs implicate that when designing S1R-targeted interventions to rescue PR cells, a potential impact on RGCs should be taken into consideration.

## Conclusions

To date, there is essentially no treatment for RP, which affects more than a million people worldwide by progressive deterioration of vision [39]. While S1R appears to be a promising pathway for intervention to protect cones that are responsible for daylight vision [23], cone death is caused by dying rods in RP [25]. It is thus important to understand the specific role(s) of S1R at different stages of PR death. Our study reveals a biphasic effect of S1R knockout on rd10 photoreceptors, first boosting rod survival, and then exacerbating apoptosis and rod loss and finally necroptosis and cone death (Fig. 10). This work may inform important implications for future development of S1R-targeted retina-protective therapeutic methods. For example, appropriate timing for delivery of a S1R-activating agent [23] or its combination with a necroptosis inhibitor [25], could produce improved therapeutic effects.

## Additional file

Additional file 1: Figure S1. Negative controls of RIP1/RIP3 proximity ligation and immunostaining. (DOCX 316 kb)

## References

[1] Hayashi T, Su TP (2007). Sigma-1 receptor chaperones at the ER-mitochondrion interface regulate Ca(2+) signaling and cell survival. Cell.

[2] Hayashi T (2015). Sigma-1 receptor: the novel intracellular target of neuropsychotherapeutic drugs. J Pharmacol Sci.

[3] Fontanilla D, Johannessen M, Hajipour AR, Cozzi NV, Jackson MB, Ruoho AE (2009). The hallucinogen N,N-dimethyltryptamine (DMT) is an endogenous sigma-1 receptor regulator. Science.

[4] Rennekamp AJ, Huang XP, Wang Y, Patel S, Lorello PJ, Cade L, Gonzales AP, Yeh JR, Caldarone BJ, Roth BL (2016). Sigma1 receptor ligands control a switch between passive and active threat responses. Nat Chem Biol.

[5] Romero L, Merlos M, Vela JM (2016). Antinociception by Sigma-1 Receptor Antagonists: Central and Peripheral Effects. Adv Pharmacol.

[6] Maurice T, Su TP (2009). The pharmacology of sigma-1 receptors. Pharmacol Ther.

[7] Nguyen L, Lucke-Wold BP, Mookerjee SA, Cavendish JZ, Robson MJ, Scandinaro AL, Matsumoto RR (2015). Role of sigma-1 receptors in neurodegenerative diseases. J Pharmacol Sci.

[8] Urfer R, Moebius HJ, Skoloudik D, Santamarina E, Sato W, Mita S, Muir KW (2014). Phase II trial of the Sigma-1 receptor agonist cutamesine (SA4503) for recovery enhancement after acute ischemic stroke. Stroke.

[9] Francardo V, Bez F, Wieloch T, Nissbrandt H, Ruscher K, Cenci MA (2014). Pharmacological stimulation of sigma-1 receptors has neurorestorative effects in experimental parkinsonism. Brain.

[10] Hedskog L, Pinho CM, Filadi R, Ronnback A, Hertwig L, Wiehager B, Larssen P, Gellhaar S, Sandebring A, Westerlund M (2013). Modulation of the endoplasmic reticulum-mitochondria interface in Alzheimer's disease and related models. Proc Natl Acad Sci U S A.

[11] Ryskamp D, Wu J, Geva M, Kusko R, Grossman I, Hayden M, Bezprozvanny I (2017). The sigma-1 receptor mediates the beneficial effects of pridopidine in a mouse model of Huntington disease. Neurobiol Dis.

[12] Mavlyutov TA, Epstein ML, Verbny YI, Huerta MS, Zaitoun I, Ziskind-Conhaim L, Ruoho AE (2013). Lack of sigma-1 receptor exacerbates ALS progression in mice. Neuroscience.

[13] Al-Saif A, Al-Mohanna F, Bohlega S (2011). A mutation in sigma-1 receptor causes juvenile amyotrophic lateral sclerosis. Ann Neurol.

[14] Luty AA, Kwok JB, Dobson-Stone C, Loy CT, Coupland KG, Karlstrom H, Sobow T, Tchorzewska J, Maruszak A, Barcikowska M (2010). Sigma nonopioid intracellular receptor 1 mutations cause frontotemporal lobar degeneration-motor neuron disease. Ann Neurol.

[15] Miki Y, Mori F, Kon T, Tanji K, Toyoshima Y, Yoshida M, Sasaki H, Kakita A, Takahashi H, Wakabayashi K (2014). Accumulation of the sigma-1 receptor is common to neuronal nuclear inclusions in various neurodegenerative diseases. Neuropathology.

[16] Schmidt HR, Zheng S, Gurpinar E, Koehl A, Manglik A, Kruse AC (2016). Crystal structure of the human sigma1 receptor. Nature.

[17] Wang J, Cui X, Roon P, Saul A, Smith SB (2017). The Role of Sigma1R in Mammalian Retina. Adv Exp Med Biol.

[18] Mavlyutov TA, Guo LW (2017). Peeking into Sigma-1 Receptor Functions Through the Retina. Adv Exp Med Biol.

[19] Smith SB, Duplantier J, Dun Y, Mysona B, Roon P, Martin PM, Ganapathy V (2008). In vivo protection against retinal neurodegeneration by sigma receptor 1 ligand (+)-pentazocine. Invest Ophthalmol Vis Sci.

[20] Zhao L, Chen G, Li J, Fu Y, Mavlyutov TA, Yao A, Nickells RW, Gong S, Guo LW (2017). An intraocular drug delivery system using targeted nanocarriers attenuates retinal ganglion cell degeneration. J Control Release.

[21] Mavlyutov TA, Nickells RW, Guo LW (2011). Accelerated retinal ganglion cell death in mice deficient in the Sigma-1 receptor. Mol Vis.

[22] Ha Y, Saul A, Tawfik A, Williams C, Bollinger K, Smith R, Tachikawa M, Zorrilla E, Ganapathy V, Smith SB (2011). Late-onset inner retinal dysfunction in mice lacking sigma receptor 1 (sigmaR1). Invest Ophthalmol Vis Sci.

[23] Wang J, Saul A, Roon P, Smith SB (2016). Activation of the molecular chaperone, sigma 1 receptor, preserves cone function in a murine model of inherited retinal degeneration. Proc Natl Acad Sci U S A.

[24] Zhao L, Zabel MK, Wang X, Ma W, Shah P, Fariss RN, Qian H, Parkhurst CN, Gan WB, Wong WT (2015). Microglial phagocytosis of living photoreceptors contributes to inherited retinal degeneration. EMBO molecular medicine.

[25] Murakami Y, Matsumoto H, Roh M, Suzuki J, Hisatomi T, Ikeda Y, Miller JW, Vavvas DG (2012). Receptor interacting protein kinase mediates necrotic cone but not rod cell death in a mouse model of inherited degeneration. Proc Natl Acad Sci U S A.

[26] Chang B, Hawes NL, Pardue MT, German AM, Hurd RE, Davisson MT, Nusinowitz S, Rengarajan K, Boyd AP, Sidney SS (2007). Two mouse retinal degenerations caused by missense mutations in the beta-subunit of rod cGMP phosphodiesterase gene. Vis Res.

[27] Zhao L, Li J, Fu Y, Zhang M, Wang B, Ouellette J, Shahi PK, Pattnaik BR, Watters JJ, Wong WT, Guo LW (2017). Photoreceptor protection via blockade of BET epigenetic readers in a murine model of inherited retinal degeneration. J Neuroinflammation.

[28] Mavlyutov TA, Epstein M, Guo LW (2015). Subcellular localization of the sigma-1 receptor in retinal neurons - an electron microscopy study. Sci Rep.

[29] Shi X, Guo LW, Seedial SM, Si Y, Wang B, Takayama T, Suwanabol PA, Ghosh S, DiRenzo D, Liu B, Kent KC (2014). TGF-beta/Smad3 inhibit vascular smooth muscle cell apoptosis through an autocrine signaling mechanism involving VEGF-A. Cell Death Dis.

[30] Hannemann L, Suppanz I, Ba Q, MacInnes K, Drepper F, Warscheid B, Koch HG (2016). Redox Activation of the Universally Conserved ATPase YchF by Thioredoxin 1. Antioxid Redox Signal.

[31] Pattnaik BR, Shahi PK, Marino MJ, Liu X, York N, Brar S, Chiang J, Pillers DA, Traboulsi EI (2015). A Novel KCNJ13 Nonsense Mutation and Loss of Kir7.1 Channel Function Causes Leber Congenital Amaurosis (LCA16). Hum Mutat.

[32] Zhao J, Ha Y, Liou GI, Gonsalvez GB, Smith SB, Bollinger KE (2014). Sigma receptor ligand, (+)-pentazocine, suppresses inflammatory responses of retinal microglia. Invest Ophthalmol Vis Sci.

[33] Wang J, Shanmugam A, Markand S, Zorrilla E, Ganapathy V, Smith SB (2015). Sigma 1 receptor regulates the oxidative stress response in primary retinal Muller glial cells via NRF2 signaling and system xc(−), the Na(+)-independent glutamate-cystine exchanger. Free Radic Biol Med.

[34] Vollrath JT, Sechi A, Dreser A, Katona I, Wiemuth D, Vervoorts J, Dohmen M, Chandrasekar A, Prause J, Brauers E (2014). Loss of function of the ALS protein SigR1 leads to ER pathology associated with defective autophagy and lipid raft disturbances. Cell Death Dis.

[35] Schrock JM, Spino CM, Longen CG, Stabler SM, Marino JC, Pasternak GW, Kim FJ (2013). Sequential cytoprotective responses to Sigma1 ligand-induced endoplasmic reticulum stress. Mol Pharmacol.

[36] Ha Y, Dun Y, Thangaraju M, Duplantier J, Dong Z, Liu K, Ganapathy V, Smith SB (2011). Sigma receptor 1 modulates endoplasmic reticulum stress in retinal neurons. Invest Ophthalmol Vis Sci.

[37] Hanus J, Zhang H, Wang Z, Liu Q, Zhou Q, Wang S (2013). Induction of necrotic cell death by oxidative stress in retinal pigment epithelial cells. Cell Death Dis.

[38] Sato K, Li S, Gordon WC, He J, Liou GI, Hill JM, Travis GH, Bazan NG, Jin M (2013). Receptor interacting protein kinase-mediated necrosis contributes to cone and rod photoreceptor degeneration in the retina lacking interphotoreceptor retinoid-binding protein. J Neurosci.

[39] Rodriguez-Muela N, Hernandez-Pinto AM, Serrano-Puebla A, Garcia-Ledo L, Latorre SH, de la Rosa EJ, Boya P (2015). Lysosomal membrane permeabilization and autophagy blockade contribute to photoreceptor cell death in a mouse model of retinitis pigmentosa. Cell Death Differ.

[40] Oyadomari S, Mori M (2004). Roles of CHOP/GADD153 in endoplasmic reticulum stress. Cell Death Differ.

[41] Sano R, Reed JC (1833). ER stress-induced cell death mechanisms. Biochim Biophys Acta.

[42] Mavlyutov TA, Yang H, Epstein ML, Ruoho AE, Yang J, Guo LW. APEX2-enhanced electron microscopy distinguishes sigma-1 receptor localization in the nucleoplasmic reticulum. Oncotarget. 2017;8:51317–30. 10.18632/oncotarget.17906 PMC5584251 28881650

