# Supplementary material for: Role of the sigma-1 receptor chaperone in rod and cone photoreceptor degenerations in a mouse model of retinitis pigmentosa
Source: Mol Neurodegener. 2017 Sep 19;12:68. doi: 10.1186/s13024-017-0202-z (PMC5606113; doi:10.1186/s13024-017-0202-z)

**Role of the sigma-1 receptor chaperone in rod and cone photoreceptor degenerations in a mouse model of retinitis pigmentosa**

Huan Yang^1,#^, Yingmei Fu^1,2,#^, Xinying Liu^3^, Pawan K. Shahi^3^, Timur A. Mavlyutov^4^, Jun Li^1,5,6^, Annie Yao^1^, Steven Guo^1^, Bikash R. Pattnaik^3,7,^*, and Lian-Wang Guo^1,7,^*

^1^Department of Surgery, Wisconsin Institute for Medical Research, University of Wisconsin School of Medicine and Public Health, Madison, WI 53705, USA

^2^Shanghai Key Laboratory of Psychotic Disorders, Shanghai Mental Health Center, Shanghai Jiao Tong University School of Medicine, 600 Wanping Nan Road, Shanghai, 200030, PR China.

^3^Department of Pediatrics, Department of Ophthalmology and Visual Sciences, University of Wisconsin, Madison, Wisconsin, USA

^4^Department of Anesthesiology, Wisconsin Institute for Medical Research, University of Wisconsin School of Medicine and Public Health, Madison, WI 53705, USA

^5^Department of Ophthalmology, the First Hospital of China Medical University, Shenyang, 110001, PR China

^6^Department of Ophthalmology, the 3rd People’s Hospital of Dalian, Dalian, 116033, PR China

^7^McPherson Eye Research Institute, University of Wisconsin, Madison, WI 53705, USA

^8^Department of Surgery and Department of Physiology &Cell Biology, the Ohio State University, Columbus, OH 43210, USA

*Short title: Sigma-1 receptor knockout in an RP model*

^#^These authors contributed equally to this work.

* Corresponding authors:

Lian-Wang Guo, Ph.D.

Department of Surgery and Department of Physiology &Cell Biology

The Ohio State University, Columbus, OH 43210, USA

Tel: +1 614 292 5276, Fax: +1 614 247 7799

Email: guo@surgery.wisc.edu

Bikash R. Pattnaik, Ph.D

Department of Pediatrics

Department of Ophthalmology and Visual Sciences

University of Wisconsin.

1300 University Avenue, SMI 112

Madison, WI 53706, USA

Tel: +1 608 265 9486

Email: [pattnaik@wisc.edu](mailto:pattnaik@wisc.edu)

**Competing Interests:**The authors have declared that no competing interests exist.

**Figure S1. *Negative controls of RIP1/RIP3 proximity ligation and immunostaining***

Retinal sections were incubated with non-specific IgG instead of primary antibodies. Shown in A-D are controls corresponding to Figure 2, Figure 3, Figure 4, and Figure 9, respectively.


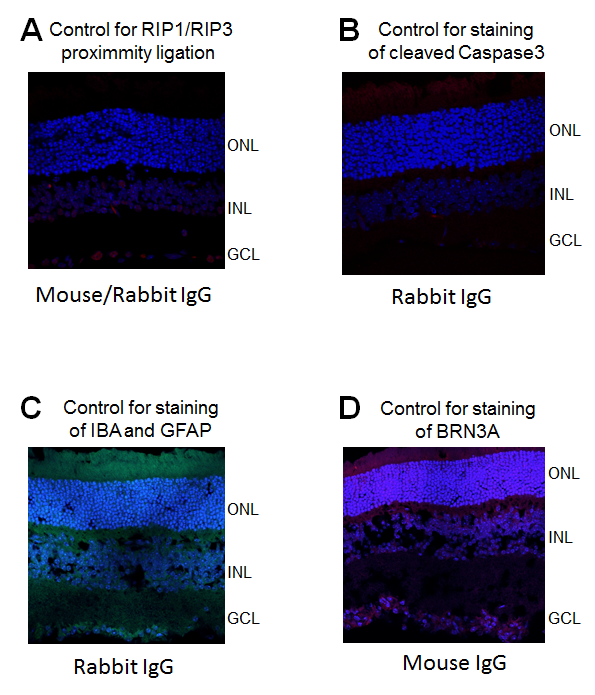

Supplement: Additional file 1: Figure S1. — Negative controls of RIP1/RIP3 proximity ligation and immunostaining. (DOCX 316 kb) [file 13024_2017_202_MOESM1_ESM.docx]
